# The Epigenetic Connection Between the Gut Microbiome in Obesity and Diabetes

**DOI:** 10.3389/fgene.2019.01329

**Published:** 2020-01-15

**Authors:** Manvi Sharma, Yuanyuan Li, Matthew L. Stoll, Trygve O. Tollefsbol

**Affiliations:** ^1^ Department of Biology, University of Alabama at Birmingham, Birmingham, AL, United States; ^2^ Department of Pharmacology and Toxicology, University of Alabama at Birmingham, Birmingham, AL, United States; ^3^ Comprehensive Cancer Center, University of Alabama at Birmingham, Birmingham, AL, United States; ^4^ Nutrition Obesity Research Center, University of Alabama at Birmingham, Birmingham, AL, United States; ^5^ Division of Pediatric Rheumatology, University of Alabama at Birmingham, Birmingham, AL, United States; ^6^ Comprehensive Center for Healthy Aging, University of Alabama at Birmingham, Birmingham, AL, United States; ^7^ Comprehensive Diabetes Center, University of Alabama at Birmingham, Birmingham, AL, United States

**Keywords:** epigenetic, gut microbiome, diet, metabolic, obesity, diabetes

## Abstract

Metabolic diseases are becoming an alarming health issue due to elevated incidences of these diseases over the past few decades. Various environmental factors are associated with a number of metabolic diseases and often play a crucial role in this process. Amongst the factors, diet is the most important factor that can regulate these diseases *via* modulation of the gut microbiome. The gut microbiome participates in multiple metabolic processes in the human body and is mainly responsible for regulation of host metabolism. The alterations in function and composition of the gut microbiota have been known to be involved in the pathogenesis of metabolic diseases *via* induction of epigenetic changes such as DNA methylation, histone modifications and regulation by noncoding RNAs. These induced epigenetic modifications can also be regulated by metabolites produced by the gut microbiota including short-chain fatty acids, folates, biotin and trimethylamine-*N*-oxide. In addition, studies have elucidated the potential role of these microbial-produced metabolites in the pathophysiology of obesity and diabetes. Hence, this review focuses on the interactions between the gut microbiome and epigenetic processes in the regulation and development of obesity and diabetes, which may have potential as a novel preventive or therapeutic approach for several metabolic and other human diseases.

## The Gut Microbiome

The gut microbiota consists of trillions of microorganisms including bacteria, archaea, viruses, and eukaryotes present in the intestine of humans (Allin et al., 2015). Generally, the gut microbiota is populated by *Bacteroidetes, Firmicutes, Actinobacteria, Proteobacteria, Verrucomicrobia, Tenericutes,* and *Lentisphaerae* as the predominant phyla. The major genera are *Pseudomonas, Streptococcus, Prevotella, Fusobacteria, Veillonella, Haemophilus, Neisseria*, *Porphyromonas, Bacteroides, Clostridium, Faecalibacterium, Eubacterium, Ruminococcus, Peptococcus, Peptostreptococcus, Lactobacillus, Streptomyces,* and *Bifidobacterium* (Marlicz et al., 2018; Neu and Pammi, 2018; Rea et al., 2018). Another similar term, gut microbiome, refers to the total genomes of gut microbiota and is often used to describe the entity of microbial functions encoded by gut microbiota (Schlaeppi and Bulgarelli, 2015). The advents in gene sequencing have revealed that the genome of human gut microbial communities (~3 million genes) is more than 100 times as large as the human genome (Gill et al., 2006), while the human:bacterial cells ratio is thought to be approximately 1:1 (Sender et al., 2016).

A neonate may procure its microbiota from the environment during delivery and also from its mother *via* breastfeeding (Dominguez-Bello et al., 2010). Studies have shown that neonates born through normal deliveries have an early and abundant composition of *Lactobacillus, Bacteroides*, and *Prevotella;* however, neonates born with cesarean deliveries have a delay in onset or lower levels of *Bacteroides, Bifidobacteria,* and *Lactobacillus* and predominately have colonization of *Clostridium difficile*, *Clostridium perfringens*, and *Escherichia coli* (Gronlund et al., 1999; Tsuji et al., 2012; Bokulich et al., 2016; Nagpal et al., 2016; Nagpal et al., 2017). These *Lactobacillus* species are known as probiotics due to their health-promoting properties and preventive properties in various metabolic diseases such as obesity and diabetes (Azad et al., 2018). However, *C. difficile* and *C. perfringens* bacteria are known for production of toxins, which can cause lethal diseases such as food poisoning, infection, diarrhea and colitis in humans (Noren, 2010).

**Figure 1:**
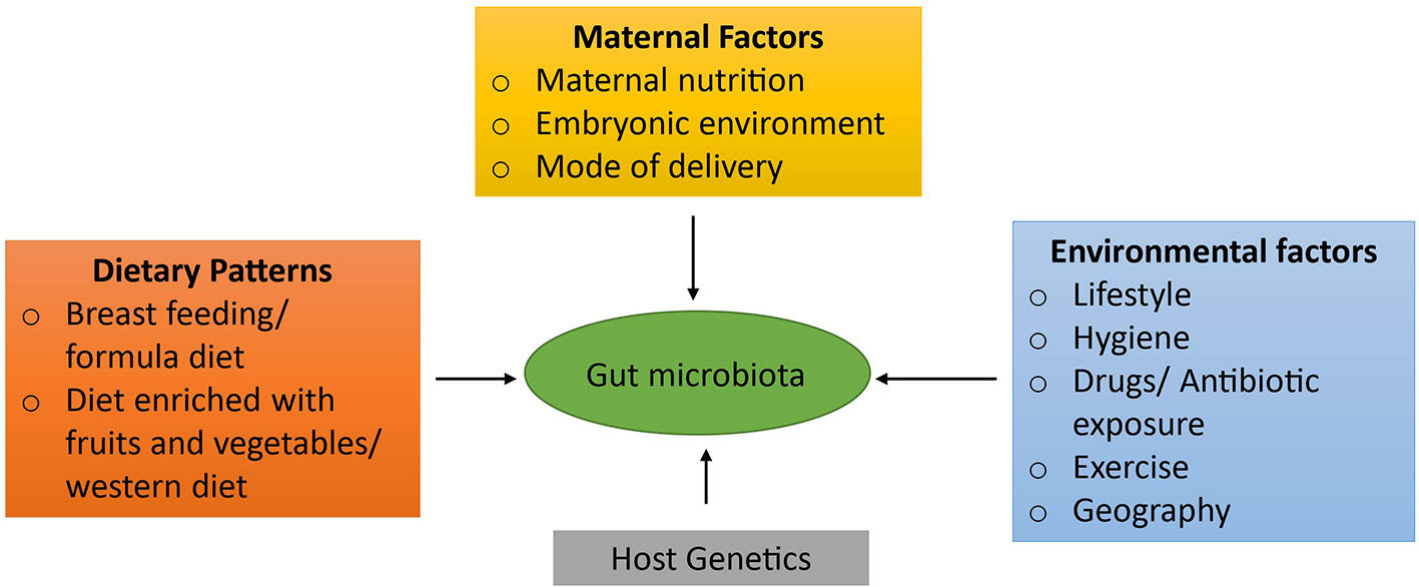
Factors influencing the development of gut microbiota. Numerous factors play a paramount role in the development of gut microbiota. Initially, the gut microbiota is acquired from a mother during the pregnancy *via* maternal nutrition, embryonic environment, and the mode of delivery. In addition, dietary patterns plays the most crucial role in maturation as well as maintenance of the gut flora such as breast feeding, formula diet, the composition of diet: diet rich in fruits and vegetables or western diet. Other factors that also contribute to its shaping are individual's genetic factors and environmental factors such as drug or antibiotic intake, infections, life style patterns, migration to a different location, etc.

During the early developmental phase, dietary factors also play a pivotal role in shaping the microbiota (Cotillard et al., 2013). For example, the gut microbiota of breast-fed neonates is mainly dominated by *Bifidobacteria*, *Lactobacillus, Staphylococcus,* and *Streptococcus* as compared to neonates that were on infant formulas, who have higher numbers of *Bacteroides*, *Clostridia,* and *Proteobacteria* (Favier et al., 2002; Bokulich et al., 2016). Various other studies have also elucidated that diet has a strong impact on maturation as well as maintenance of the gut microbiome (De Filippo et al., 2010; David et al., 2014) and potentially on the health of an individual (Camilleri et al., 2019). In addition to dietary factors, other potential variables such as antibiotic intake and infections can also influence gut microbiota (Qin and Wade, 2018) and can result in the disproportion and reduction of microbial biodiversity which is known as gut dysbiosis. These factors influencing the development of the gut microbiota are depicted in **Figure 1**.

A newborn infant's microbiota starts to develop according to dietary and environmental factors, but lacks an abundant diversity of commensal microorganisms. Over the first few years of life, the young child's microbiota undergoes substantial alterations, ultimately transitioning to a more mature pattern by age 3 or 4, by which time the pace of change slows down (Koenig et al., 2011; Yatsunenko et al., 2012; Stewart et al., 2018). During adulthood, in the absence of major environmental perturbations, the microbiota appears to be stable for many years, perhaps the whole lifespan (Mehta et al., 2018). These microbiota and their functions represent a “core gut microbiome,” which consists of particular types of gut microbial species, which resides in every individual and is mainly responsible for the correct functioning of a gut microbial ecosystem (Turnbaugh and Gordon, 2009; Qin et al., 2010). Gut commensal communities impact several metabolic functions within the human body such as digestion, nutrients absorption, regulation of intestinal hormones secretion, modulation of intestinal immunity and inflammatory processes, synthesis of vitamins, amino acids, and various metabolites such as short-chain fatty acids (SCFAs), choline, and lipids (Nicholson et al., 2012; Allin et al., 2015; Devaux and Raoult, 2018). In addition to the metabolic functions, the gut microbiome also shapes gene expression in the host. Through various microbial-derived metabolites, gut bacteria can influence the host metabolism by inducing epigenetic alterations of key genes which modulate the initiation and progression of diseases (Yuille et al., 2018). Therefore, gut dysbiosis has been associated with an increasing incidence of conditions such as metabolic diseases such as type 2 diabetes (Allin et al., 2015) and obesity (Ley et al., 2006); inflammatory diseases such as inflammatory bowel disease (Pascal et al., 2017) and rheumatoid arthritis (Maeda and Takeda, 2017); liver diseases (Janssen et al., 2017); and cancers (Lofgren et al., 2011; Yu et al., 2017).

With the increasing prevalence of metabolic disorders such as obesity and diabetes in Western countries, it is essential to focus on the interplay between epigenetic mechanisms and gut microbial composition in the induction of these diseases that might provide a novel therapeutic approach for prevention and treatment. Hence, we will discuss the potential roles of the gut microbiome and epigenetics in the pathophysiology and pathogenesis of obesity and diabetes (type 1 and type 2).

## The Gut Microbiome and Epigenetics

Epigenetics is the study of phenotypic changes secondary to alterations in gene expression that do not directly arise from changes in the underlying DNA sequence. In eukaryotes, epigenetic mechanisms primarily involve DNA methylation, posttranscriptional histone modifications, chromatin restructuring, and regulation of gene expression by noncoding RNAs (Paul et al., 2015). These epigenetic mechanisms can be regulated by cross-talk of microbial metabolites, external factors such as diet, antibiotics and also by other environmental factors (pH, oxygen, and temperature), resulting in the modulation of a large number of human metabolic diseases (Romano and Rey, 2018).

### Noncoding RNA

The noncoding RNA (ncRNA) are RNA transcripts that are not translated into the proteins (Dempsey et al., 2018). The alterations in ncRNA have been known to contribute to various diseases such as obesity, diabetes, neurodegenerative diseases, liver diseases, and lung diseases (Devaux and Raoult, 2018). The major types of ncRNAs are microRNA (miRNA), short-interfering RNAs (siRNAs), piwi-interacting RNAs (piRNAs), and long noncoding RNAs (lncRNAs). lncRNAs modify gene expression *via* the formation of complexes with chromatin-modifying protein and by functioning as signaling and guide molecules (Devaux and Raoult, 2018). A few studies have focused on the role of the gut microbiome in regulation of lncRNA gene expression in the host. In order to obtain in-depth knowledge about their correlation, Liang et al. performed a bioinformatics study on characterization of lncRNA regulated by gut microbiome in intestinal epithelial tissues of mice (Liang et al., 2015). The comparison between germ-free mice with the conventional (re-colonized with mice microbiota) and gnotobiotic mice (recolonized with either *E. coli* or *E. coli*-expressing bile salt hydrolase) showed that only six lncRNAs were commonly upregulated. These lncRNAs were also found to be overexpressed in immune organs such as thymus and spleen, which may reflect their crucial role in immune processes. Furthermore, this study also elucidated that lncRNA expression profiles successfully differentiated gontobiotic mice from conventional mice based on their gut microbial composition. Despite the less available information on the association of lncRNA and gut microbiota, this study provided novel insight of microbial-regulated lncRNA expression. This study may also enhance understanding of the potential roles played by lncRNA expression that are regulated by gut microbiota and in-turn influence metabolic disorders such as obesity and diabetes. However, this study had a limitation with respect to determining whether the expression of lncRNAs was regulated by the gut microbial communities of the host (Liang et al., 2015). Another study by Dempsey et al. evaluated lncRNA expression in various tissues- liver, duodenum, jejunum, ileum, white adipose tissue, brown adipose tissue, colon, and skeletal muscle from germ-free and conventional mice (Dempsey et al., 2018). They found, in the absence of microbial communities (i.e., germ-free mice), the lncRNAs were differentially regulated in distal (liver, muscle, and fat) tissues as well as in proximal (intestinal) tissues. Most of the lncRNAs which were found to be regulated by gut microbiome were present in an essential metabolic organ, namely, the jejunum. Overall, this is the first study that demonstrated that the gut microbiome is important for the lncRNA expression in the various metabolic and other organs (Dempsey et al., 2018). These studies implicate a potential role played by the gut microbiome in regulation of lncRNA expression. However, more investigations in this context should be conducted to further clarify the role of this association of host-microbe interactions with the pathogenesis of obesity and diabetes.

Another type of ncRNA, miRNAs, also play crucial roles in maintaining metabolic homeostasis and in development of obesity and insulin resistance (Davalos et al., 2011; Trajkovski et al., 2011), This has also been shown in a recent study by Virtue et al. that focused on the interplay between miRNAs and development of obesity through gut microbial population in germ-free and conventionally housed specific pathogen-free mice (Virtue et al., 2019). The investigators found that the *miR-181a* and *miR-181b* expression were increased in epididymal white adipose tissues obtained from conventional mice as compared to germ-free mice, which implicates the importance of gut microbiota in the regulation of miRNA. In order to confirm this regulation, they colonized germ-free mice with conventional mice microbiota and found similar results, that is, upregulation of *miR-181a* and *miR-181b* in epididymal white adipocytes. The investigators hypothesized that the potential mechanism behind the regulation of *miR-181* might be *via* gut microbial-produced metabolites. This leads to the finding that tryptophan-derived metabolites negatively regulate *miR-181* expression in white adipocytes, which further influence the pathways of adiposity, energy balance and insulin sensitivity. In addition, this study also revealed the imbalance of the microbiota—*miR-181* axis is vital for obesity and insulin resistance development (Virtue et al., 2019). Therefore, this novel study elaborates that the gut microbiota might serve as a key regulator for functioning of miRNAs especially in white adipocyte tissue, which have a major impact on the development of obesity and insulin resistance. However, this study has not provided the role of specific gut microbial species that may be critically involved in the regulation of *miR-181* expression. Further well-designed studies are required for elucidating the role of the gut microbiome on miRNA in the etiology of obesity as well as in diabetes.

### DNA Methylation

DNA methylation refers to the inclusion of a methyl group (-CH_3_) on the carbon-5 of the cytosine ring in cytosine-guanine dinucleotide-rich (CpG) regions (Romano and Rey, 2018). The enzymes, DNA methyltransferases (DNMTs) catalyze this process and also regulate the gene expression. These DNMTs are highly sensitive to the availability of nutrients that can also be affected by the metabolic activities of the microbial species present in the gut (Romano and Rey, 2018). The major metabolic activity involves synthesis of metabolites that can modulate the epigenome by participating in one-carbon metabolism (Mischke and Plosch, 2013). Metabolites such as folate, vitamin B12, betaine, and choline are potentially involved in the synthesis of 6-methyltetrahydrofolate, which is a methyl group donor, for the generation of S-adenosylmethionine (SAM) that participates in DNA methylation processes (Kovacheva et al., 2007; Crider et al., 2012; Kok et al., 2015; Zeisel, 2017; Mahmoud and Ali, 2019). These methyl donor nutrients are found to be regulated by specific gut microbial communities such as *Lactobacillus* and *Bifidobacteria* that are known for folate production (Strozzi and Mogna, 2008; Rossi et al., 2011). In order to understand the role played by *Lactobacillus* and *Bifidobacterium* in regulation of diabetes, a study by Murri et al. found their levels were reduced in type 1 diabetic healthy Caucasian children (Murri et al., 2013). These studies show an important association between gut microbial communities and DNA methylation mechanisms that may regulate diabetes.

Other crucial metabolites, such as SCFAs are synthesized in the gut by the fermentation of nondigestible carbohydrates by certain microbes. Some of the major SCFAs including butyrate can also influence DNA methylation processes by inducing phosphorylation of *ERK* (MAP kinase1), which results in down-regulation of DNMT1 and consequently demethylation of tumor suppressor genes including *RARB2*, *p21,* and *p16* (Sarkar et al., 2011). The amount of butyrate synthesis depends on both dietary intake and synthesis by gut microbial communities. Various bacteria are known for butyrate production including *Megasphaera, Odoribacter, Eubacterium, Peptoniphilus, Fusobacterium, Coprococcus, Porphyromonas, Faecalibacterium, Anaerotruncus, Clostridium, Subdoligranulum,* and *Roseburia* (Demehri et al., 2016). The butyrate-producing bacteria, *Clostridium,* was found to be present in abundant levels in p21-*p*-luc male mice, which were treated with a high-fat diet (HFD) diet for thirty weeks when compared with mice on a normal diet (Yoshimoto et al., 2013), indicating the potential role of diet in impacting the gut microbiota and downstream metabolic processes. Similarly, the number of *Clostridium* was increased in type 1 diabetic children (Murri et al., 2013). These studies suggest an important role by gut microbiota in regulation of obesity and diabetes *via* production of gut metabolites.

Several other crucial nutrients also play a vital role in the regulation of the DNA methylation process (Liu et al., 2019). For instance, choline is a water-soluble vitamin-like nutrient found in various food sources such as meat, grain, milk, egg, and their derived products. Gut communities can metabolize choline into several metabolites that impact human health, such as trimethylamine (TMA) (Romano et al., 2015). TMA can be further metabolized by flavin monooxygenase (FMO) enzymes into trimethylamine-*N*-oxide (TMAO) (Baker and Chaykin, 1960), which has been linked to obesity and diabetes (Fennema et al., 2016; Velasquez et al., 2016; Tang and Hazen, 2017). This choline derived metabolite, TMAO was also found to be involved in vast production of ROS (Sun et al., 2016), that in-turn can influence epigenetic programming as it can lead to deamination or depurination of nucleic acids, which may trigger DNA repair mechanisms and replacement with a nonmethylated cytosine (Avila et al., 2015). To investigate the relationship between choline and metabolic disorders *via* DNA methylation, a study was conducted on germ-free C57BL/6 female mice (Romano et al., 2017). These mice were divided into two different groups based on the colonization in gut; a) with choline-utilizing bacteria, or b) with bacteria unable to utilize choline and unable to produce TMA. Both mouse groups were kept on HFD supplemented with choline for several weeks, which resulted in lower methylation levels in heart, colon, brain, and liver tissues in the first group of mice as compared to the other group. In addition, the mice colonized with choline-utilizing bacteria displayed adiposity features. It was concluded that choline-utilizing bacteria compete for choline uptake with the host, resulting in lower levels of choline and methyl donor in the host, ultimately making the host more susceptible toward metabolic disorders (Romano et al., 2017). These studies implicate the association of choline in modulating epigenetic machinery that might act as a key player in the pathophysiology of metabolic diseases. However, more studies are required to confirm the exact mechanisms induced by choline on modulating the DNA methylation process.

### Histone Modifications and Chromatin Remodeling

Histones are proteins that are wound by DNA in the nucleus to form the condensed chromatin and mainly comprise of four families: H1, H2A, H2B, H3, and H4. Histones are prone to various modifications including acetylation, methylation, phosphorylation, SUMOylation, poly-ADP ribosylation, biotinylation, ubiquitination, citrullination, and proline isomerization (Bernstein et al., 2007; Paul et al., 2015). Out of all modifications, histone methylation, acetylation, and deacetylation are known to play key roles in the induction and progression of various disorders. The histone methylation process involves the addition of methyl groups to the histone proteins by histone methyltransferases (HMTs) enzymes. Histone methylation can either lead to transcription activation (e.g., H3K4) or transcription inactivation (e.g., H3K9, H3K27), depending on the specific residue and modifications (Orouji and Utikal, 2018). A recent study by Tateishi et al. showed a correlation of histone methylation with obesity and hyperlipidemia in mice. They found that impairment in Jhdm2a function (a H3K9-specific histone demethylase) in *Jhdm2a* knockout mice resulted in altered β-adrenergic-stimulated glycerol release and oxygen absorption in brown fat. This study also revealed that β-adrenergic activation induces Jhdm2a binding to the PPAR responsive element of the *Ucp1* gene, an important gene involved in energy balance and that leads to a decrease in H3K9me2, which contributed to obese phenotypes such as fat deposition and rise in lipid content (Tateishi et al., 2009).

Histone acetyltransferases (HATs) enzymes catalyze the transfer of acetyl groups from acetyl-CoA to the amino-terminal lysine residues on histone proteins (Roth et al., 2001). The histone acetylation process can be regulated by various gut-microbial derived metabolites such as SCFAs (Krautkramer et al., 2016; Qin and Wade, 2018). It has been found that supplementation with acetate raised the acetylation levels of brain histones H3 at lysine 9 and H4 at lysine 8 and 16 that resulted in neuroglial activation and decline in the cholinergic cell (a nerve cell) in a rat model of LPS-induced neuroinflammation (Soliman and Rosenberger, 2011). A recent study by Wang et al. investigated the impact of lentinan, a polysaccharide derived from mushroom, on the intestinal microbiota of piglets that were challenged with *E. coli* lipopolysaccharide-induced intestine injury. They found that lentinan supplementation increased SCFAs levels including butyrate, propionate, iso-butyrate, and isovalerate in the cecum, which further led to a rise in H3 histone acetylation and a decline in intestinal inflammation (Wang et al., 2019b). It has been evident that the chromatin state of several tissue constituents such as colonic cells, can be modulated by SCFAs produced in the gut (Krautkramer et al., 2016).

Histone deacetylases (HDACs) constitute a class of enzymes that remove an acetyl group from the amino-terminal lysine residues of histones resulting in compacted chromatin (Yuille et al., 2018). Histone deacetylation is primarily associated with transcriptional inactivation and overexpression of HDACs and has been linked to a number of neurological and inflammatory diseases. Overall, 13 HDACs have been found in humans, which are classified into four classes- Class I contains HDACs 1, 2, 3, and 8; Class IIa consists of HDACs 4, 5, 7, and 9; Class IIb contains HDACs 6 and 10; Class III is comprised of Sirt1-Sirt7 and Class IV consists of HDAC 11. Each of these plays an important role in cell survival, proliferation and differentiation which can also influence tumorigenesis (Yuille et al., 2018). HDAC inhibitors have well-known potential to act as therapeutic agents in various diseases. The gut microbiome can modulate the activity of HDACs *via* production of epigenetic metabolites such as the SCFAs. Butyrate and propionate have been identified as potential contributors to HDAC inhibition (Marlicz et al., 2018). Butyrate is essential for maintaining homeostasis in the gut and is also important in the regulation of many processes such as epigenetic mechanisms, lipogenesis, gluconeogenesis, and inflammatory conditions. Among various epigenetic modifications, butyrate is specifically known as a class I and class II HDAC inhibitor (Marlicz et al., 2018). An interesting study focused on 79 distinct commensal human gut bacteria to investigate the connection between SCFA profiles and HDAC inhibitory properties. These findings revealed that three butyrate-producing bacterial strains: *Megasphaera massiliensis* MRx0029, *Roseburia intestinalis* MRx0071, and *Bariatricus massiliensis* MRx1342, manifested the highest inhibition of HDAC activity. In addition, *M. massiliensis* produced significant levels of valeric acid and hexanoic acid, which are medium-chain fatty acids. It was also reported that valeric acid and butyrate cumulatively showed inhibition against Class I HDACs- HDACs1, 2, 3 8, particularly HDAC2 (Yuille et al., 2018).

## Gut Microbiome, Epigenetics, and Obesity

Obesity is a leading disorder that involves accumulation of excessive fat in the body. Obesity has been found to be linked with multiple conditions including cardiovascular diseases, diabetes, metabolic disorders, and cancers (Kopelman, 2000). There are various factors that have been shown to play a key role in the pathophysiology and pathogenesis of obesity such as genetic susceptibility, dietary patterns, ethnic differences, antibiotic intake, and environmental factors.

### Dietary Patterns

Diet is a vital factor in the establishment of the composition of gut microbiota, which cross-talks with the intestine, and participates in the generation of signals to communicate with distal organs such as the liver. It therefore plays an integral role in shaping the host's metabolism (Schroeder and Backhed, 2016). Differences in dietary patterns can lead to alterations in the composition and function of microbial communities, resulting in change in fermentation processes, energy consumption, sensation, and permeability in a manner that results in weight gain (Kumar et al., 2014; Devaux and Raoult, 2018). Beside these, recent evidence suggests that diets strongly influence epigenetic processes that are linked to obesity development (Qian et al., 2017). Numerous studies have indicated that the shifts in microbial communities mainly due to consumption of a “Western” diet, which is high in fat and carbohydrate, result in predisposition toward obesity. Specifically, a decrease of Bacteroidetes and an increase of Firmicutes levels have been linked with the consumption of a western diet (Ley et al., 2006). These bacterial communities have been known to directly influence the epigenetic reprogramming *via* DNA methylation. As an illustration, Kumar et al. reported that the infants born from mothers who had a higher Firmicutes gut composition showed altered DNA methylation as compared to infants born from mothers with higher composition of Bacteroidetes in the gut. This study also reported that the differentially methylated genes of infants whose mothers had high levels of Firmicutes were positively correlated to cardiovascular diseases, inflammation, obesity, and abnormal lipid metabolism (Kumar et al., 2014).

In contrast, studies have shown that certain plant-based diets have been linked to a particular spectrum of bacteria that ameliorates development of obesity. For example, a recent study focused on the impact of ginger on gut microbiota and prevention of obesity in C57BL/6J mice (Wang et al., 2019a). The mice were divided into four groups based on the feeding of normal diet or HFD diet with or without ginger. It was found the mice on the HFD and ginger diets showed a decrease in weight, as well as diminished low-grade inflammation and insulin resistance. The microbiota profile associated with these changes included abundance of *Bifidobacterium* genus and major SCFA-producer bacteria such as *Alloprevotella* and *Allobaculum*. To address whether these gut microbial differences were responsible for the metabolic improvements, the investigators performed fecal microbiota transplantation into mice whose microbiota was depleted by antibiotics. The transplants were technically successful, insofar as they recapitulated the microbiota profiles of the donor mice. Importantly, the mice that received microbiota from the HFD and ginger diet group showed a decrease in body weight, body mass and improvement in glucose tolerance as compared to recipients of microbiota from the HFD group. This study confirms the association of particular microbial species with the ginger-supplemented diet and provides an underlying mechanism of prevention of obesity *via* these microbial species (Wang et al., 2019a). Multiple other species of bacteria may beneficially impact metabolic processes. For example, *Bifidobacterium* has been known to impact metabolic events such as insulin resistance, low-grade inflammation, and obesity in mice (Zhang et al., 2017). Similarly, the SCFA-producers *Alloprevotella* and *Allobaculum* have been found to be associated with improvement in obesity and insulin resistance (Zhang et al., 2015). Also, the SCFAs produced from these bacteria such as butyrate and propionate are known to strongly influence molecular pathways that impact obesity development (Lin et al., 2012). The major SCFAs such as butyrate, propionate and acetate bind to G-protein coupled receptors (GPRs) - GPR41 (Free Fatty Acid Receptor 3) and GPR43 (Free Fatty Acid Receptor 2) expressed on the intestinal mucus layer of immune cells, liver cells, and adipose tissue (Brahe et al., 2013), resulting in decrease in weight gain, less intake of food and liposysis inhibition in adipose tissues (Lu et al., 2016). In addition to these, various other studies have focused on changes in intestinal composition with obesity and its role in metabolic mechanisms and epigenetics as highlighted in **Table 1**. Therefore, diets may act as a bridge that links the gut microbiome and host metabolism and that contributes to altered health outcomes through, at least in part, regulation of epigenetic mechanisms.

**Table 1:** The alterations in gut microbiota in obesity and its role in metabolic mechanisms and epigenetics.

Study design	Method	Gut microbiota profile	Associated metabolic mechanisms	Association between microbiota and epigenetic modifications	References
Comparison of gut microbiota between obese and lean individuals. Furthermore, twelve obese individuals were randomly categorized to either fat-restricted or carbohydrate-restricted low calorie diet	16S rRNA gene sequencing of stool samples	Decrease of *Bacteroidetes* and increase of *Firmicutes* levels in obese individuals as compared to lean people After dietary treatments, increase in *Bacteroidetes* and decrease in *Firmicutes* levels were observed in both types of diets	*Bacteroidetes* are found to be correlated with intake of energy and fat (monounsaturated, polyunsaturated, and saturated fat)	Hypomethylation and upregulation of *HDAC7* and *IGF2BP2* in adipose tissue were associated with lower *Bacteroidetes* to *Firmicutes* ratio group when compared with high *Bacteroidetes* to *Firmicutes* ratio group	(Ley et al., 2006) (Mendez-Salazar et al., 2018) (Ramos-Molina et al., 2019)
Analysis of gut microbiome diversity and richness of undernourished (n = 12), obese (n = 12), and normal weight (n = 12) Mexican school-age children	16S rRNA gene sequencing of fecal samples	Decrease in bacterial richness and diversity in undernourished and obese with comparison to normal weight group. In addition, abundant levels of *Lachnospiraceae* family and Firmicutes phylum in undernourished children than obese children. Rise in levels of Proteobacteria phylum and *Bilophila* in obese group	*Lachnospiraceae* abundance associated with leptin and negatively associated with energy intake	*Firmicutes* dominant gut microbiota showed differential methylation profile of gene promoters, associated with obesity and lipid metabolism	(Mendez-Salazar et al., 2018) (Kumar et al., 2014)
The p21-*p*-luc male mice (n = 19) were treated with dimethylbenz(*a*)anthracene, and then fed either normal or HFD diet for thirty weeks	Meta 16S rRNA gene sequencing	Abnormal rise in the levels of gram-positive bacteria, specifically Clostridium genus in mice on HFD	Significant amounts of deoxycholic acid (DCA), a gut metabolite, were found in HFD-fed mice	DCA has been known to be involved in DNA damage. The enterohepatic circulation of DCA resulted in development of obesity-associated hepatocellular carcinoma in mice	(Yoshimoto et al., 2013)

**Figure 2:**
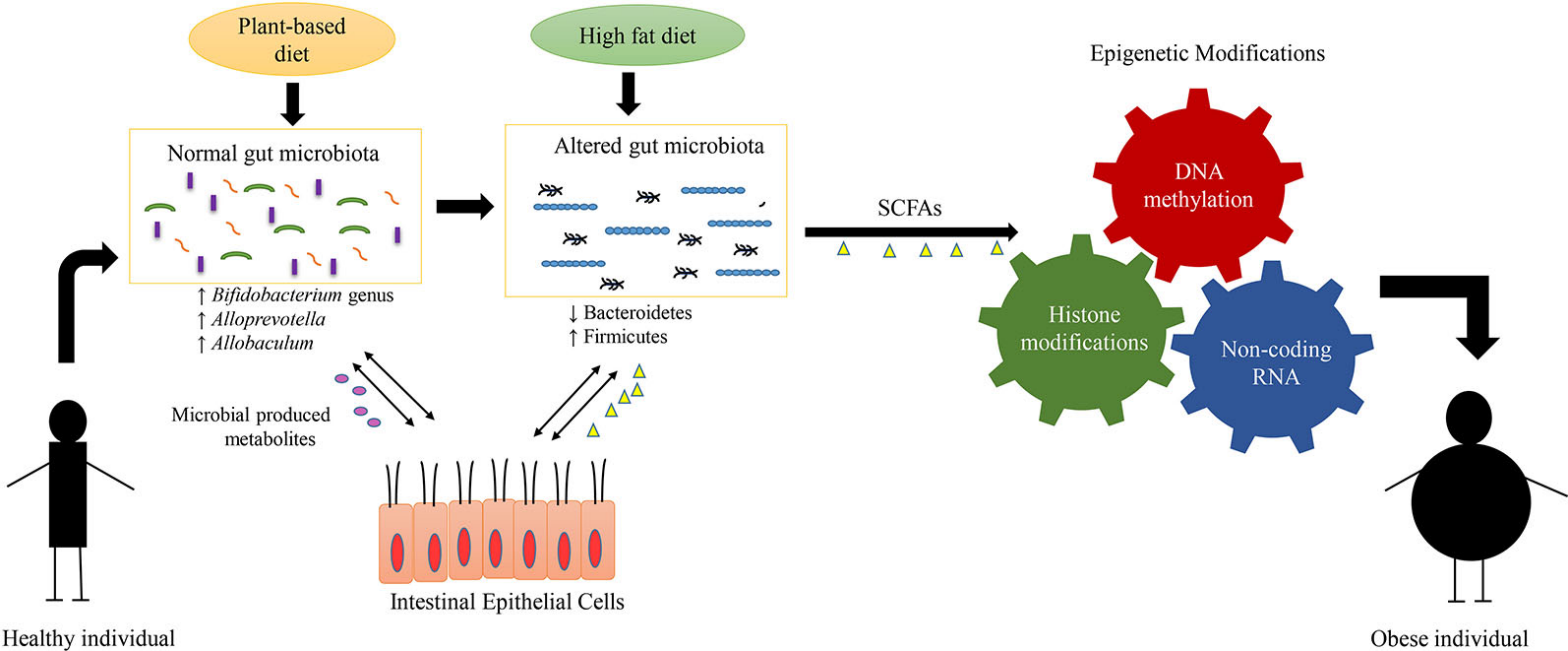
Interplay between diet, gut microbiome, epigenetic mechanisms, and obesity. The alterations in composition of microbial communities mainly arise due to difference in dietary patterns. The plant-based diets have been known to be associated with diverse and particular gut flora such as *Bifidobacterium* genus, *Alloprevotella,* and *Allobaculum*. The diverse composition of gut microbiota results in production of various metabolites such as short-chain fatty acids (SCFA). These gut microbial-produced metabolites interact with the epithelial cells of the host and help to maintain the host metabolism. On the other hand, the diets enriched in fat and carbohydrate, result in lowered gut diversity and alterations in the composition of gut microbiota such as decrease in levels of Bacteroidetes and increase in levels of Firmicutes. This gut dysbiosis (alterations in composition and function of gut bacteria) produce metabolites that induce specific epigenetic alterations such as DNA methylation, histone modification and noncoding RNA, which in-turn regulate the development of obesity.

The direct association between diet, gut bacteria, and epigenetic factors that leads to obesity development have been highlighted in **Figure 2**. Epigenetic mechanisms such as histone modifications and DNA methylations play a key role in the development of obesity (Qian et al., 2017; Lieber et al., 2018). As a study found that loss of HDAC6 has been associated with a rise in acetylation of a protein called “cell death–inducing DNA fragmentation factor subunit α-like effector C,” which further led to an increase in lipid droplet storage and ultimately body weight gain (Qian et al., 2017). DNA methylation also plays a promising role in regulation of obesity; this was demonstrated in a study involving administration of *Lactobacillus rhamnosus* and *Bifidobacterium lactis* to pregnant women, which resulted in a decrease in the methylation of the *FTO* and *MC4R* promoters in the women and their children (Vahamiko et al., 2019). The significance of these findings is that these genes such as *FTO* has been associated with weight gain and BMI; and is well-known risk factor of obesity (Claussnitzer et al., 2015; Qi et al., 2015). Also, *MC4R* gene involves in the key metabolic processes such as regulation of food consumption and energy balance; and the abnormalities in this gene can lead to decrease in satiety and onset of obesity (Fani et al., 2014; Rovite et al., 2014). Overall, this study has provided an important insight that supplementation of probiotics during pregnancy can modulate DNA methylation of promoter region of genes that are associated with weight gain and obesity in mothers and their children (Vahamiko et al., 2019). In the future, more research will be required to elaborate on the interaction between the gut microbiome and DNA methylation modifications that may play key roles in regulation of obesity.

### Inflammation

Several studies have implicated the interplay between inflammation and obesity. For instance, the inflammatory cytokines such as interleukin 1, interleukin 6, tumor necrosis factor, and C-reactive protein were found to be associated with the obesity markers- BMI, waist circumference, or percentage body fat (Marques-Vidal et al., 2012). Obesity is also associated with inflammatory conditions, such as juvenile psoriatic arthritis. These individuals were found to have a greater risk of obesity as compared to healthy children as well as children with other forms of juvenile arthritis. (Samad et al., 2018). Low-grade inflammation may contribute significantly to obesity development as high levels of activated CD8^+^T cells and aggravated immune response were observed in adipose tissue of HFD-fed mice (Nishimura et al., 2009). Regulatory T (T_reg_) cells, a subpopulation of T-helper cells, maintains balance between proinflammatory and antiinflammatory immune responses. Studies have shown dependency of T_reg_ cell on the intestinal communities as they acquire signals from the gut microbiota (Romano-Keeler et al., 2012). Forkhead box P3 (Foxp3) is a transcription factor expressed by T_reg_ cells, that plays a crucial role in the development and function of T_reg_ cells (Hori et al., 2003). This Foxp3 protein is regulated posttranslationally by lysine acetylation by HDACs and HDAC inhibition (Wang et al., 2015). Therefore, the products of gut microbiota such as SCFA, which act as HDAC inhibitors, may be involved in T_reg_ cell differentiation and ameliorate inflammatory states such as obesity. For example, butyrate plays an essential role in the differentiation of T_reg_ cells *via* increasing the acetylation of noncoding regions of the *Foxp3* locus (Furusawa et al., 2013). It specifically acts as a HDAC inhibitor in intestinal epithelial cells and thereby alters metabolic functions. For example, HDAC3 knockout mice did not show obesity features (improvement in glucose tolerance and insulin levels) despite being on the same HFD as wild-type C57BL6 mice. Further analysis of intestinal epithelial cells from HDAC3 knockout mice showed altered expression of *Chka*, *Mttp*, *Apoa1,* and *Pck1* (an enzyme involved in gluconeogenesis and glyceroneogenesis processes in adipose (Franckhauser et al., 2002)), which are associated with multiple metabolic processes in the intestinal epithelial cells. Thereafter, butyrate supplementation in control mice resulted in weight loss and refinement of metabolic functions (Whitt et al., 2018).

## Gut Microbiome, Epigenetics and Diabetes Mellitus

From the past decade, there has been a significant increase in the incidence of Diabetes (Zhou et al., 2016). Type 1 diabetes is an autoimmune disease that arises when T-cell mediated destruction of insulin-producing β cells occurs (Gianani and Eisenbarth, 2005), whereas type 2 diabetes is a chronic disease which commences when insulin resistance develops in the body (Chen et al., 2011). Due to acquired insulin resistance, more insulin, which is produced by the pancreas, is required. However, the pancreas fails to generate enough insulin, which in turn increases the blood glucose level (Larsen et al., 2010). A large number of factors such as environmental, genetic and lifestyle factors influence both type 1 and type 2 diabetes (Qin et al., 2012). Various studies have provided evidence of a direct relationship between epigenetic mechanisms and the gut microbiome in the etiology of both types of diabetes (Allin et al., 2015).

Recent experimental data from human studies have shown that the gut microbial composition has a crucial role in the development of type 1 diabetes (Brown et al., 2011; Giongo et al., 2011). As also highlighted in **Table 2**, the study by Giongo et al. focused on the children at high risk for developing type 1 diabetes based upon at-risk HLA types (Giongo et al., 2011). They were followed prospectively for the development of antibodies associated with diabetes and the microbiota of children who ultimately developed antibodies was compared to that of the children who did not develop antibodies. They found that prior to disease onset, there were alterations in gut microbiota such as reduction in Firmicutes and increase in Bacteroidetes and decreased diversity in the infants who produced antibodies (Giongo et al., 2011). However, whether the observed microbial difference arose due to differences in dietary patterns of children is not yet known. Similarly, another study by Murri et al. focused on the gut microbial profile of children diagnosed with type 1 diabetes and healthy children (Murri et al., 2013). The results of this study revealed increased levels of *Bacteroidetes*, *Clostridium* spp. and *Veillonella* as well as decline in *Lactobacillus*, *Bifidobacterium*, *Blautia*, and *Prevotella* in diabetic children when compared with healthy children. These alterations in gut microbiota were independent of dietary patterns, as no significant difference between the dietary habits was observed between both groups of children (Murri et al., 2013). The resulting limited gut microbial diversity creates an imbalance of the microbial ecosystem and essential processes in the gut which may reduce the diverse diet digestion capacity that eventually leads to a decrease in energy levels in type 1 diabetes patients and cause them to be more prone toward diseases (Giongo et al., 2011). The knowledge acquired from this study may allow interventions that can cure or delay autoimmunity in patients by changing the gut microbiota through epigenetic regulations.

**Table 2:** Role of the gut microbiome in induction of type 1 diabetes and the association between gut microbiota, metabolic mechanisms, and epigenetic modifications.

Study design	Method	Gut microbiota profile	Associated metabolic mechanisms	Association between microbiota and epigenetic modifications	References
The fecal samples were obtained from Finnish children (n = 8), before the development of antibodies associated with type 1 diabetes, at three different time intervals	16S rRNA gene sequencing of stool samples	Reduction in levels of Firmicutes and increase of Bacteroidetes in diabetic children as compared to healthy children	Bacteroidetes play a role in polysaccharides metabolism	This ratio has been linked with lower levels of methylation in *TLR 2* promoter region and in the first exon of *TLR 4*	(Giongo et al., 2011) (Mahowald et al., 2009) (Remely et al., 2014)
Analyses of fecal bacterial composition of type 1 diabetic (n = 16) and healthy Caucasian children (n = 16)	PCR-denaturing gradient gel electrophoresis and qPCR	Increased levels of *Bacteroidetes*, *Clostridium* spp. and *Veillonella*; decline in *Lactobacillus*, *Bifidobacterium*, *Blautia*, and *Prevotella* in diabetic children	*Lactobacillus* and *Bifidobacterium* were found to be associated with excessive levels of plasma glucose in diabetic children *Bifidobacterium* have also been associated with improved metabolism of glucose, low-grade inflammation, and insulin resistance	*Bifidobacterium* was associated with metabolic mechanisms and improvement in insulin resistance and low-grade inflammation. It was also associated with lowering the incidence of autoimmune diseases in genetically predisposed individual	(Murri et al., 2013) (Philippe et al., 2011) (Eslami et al., 2019) (Zhang et al., 2017) (Mu et al., 2017)
Four cohort of participants with or without islet autoimmunity residing in the U.S.	16S rRNA gene sequencing	Elevation in *Bacteroides* and *Akkermansia* and decrease in abundance of *Prevotella*	*Akkermansia* is a known acetate and propionate producing bacteria *Prevotella* are SCFAs producing bacteria and associated with long-term consumption of dietary fiber	*Akkermansia* were found to decrease the expression of *Gpr43* and *Pparγ* and increase the expression of *Hdac3* and *Hdac5*	(Alkanani et al., 2015) (Mojica and de Mejia, 2015) (Lukovac et al., 2014)
Fecal bacteria investigation of type 1 diabetic (n = 15) and healthy (n = 15) Chinese children	16S rRNA gene sequencing	Increased composition of *Blautia* and decreased composition of *Lachnospira*, *Dialister*, *Haemophilus,* and *Acidaminococcus* in diabetic children	*Blautia* breakdowns undigested proteins and carbohydrates into acetic acid, which may generate energy in the human body	Cpf1 proteins from *Acidaminococcus* and *Lachnospiraceae* showed significant genome-editing efficacy to Cas9	(Qi et al., 2016) (Li et al., 2017)

Additional evidence has shown that the intestinal microbiota community is also associated with type 2 diabetes. As highlighted in **Table 3**, a study in the Chinese population revealed a decrease in the *Roseburia* and *Faecalibacterium* species and increase in *Escherichia coli* in type 2 diabetes patients as compared to nondiabetic control subjects (Qin et al., 2012). Both *Roseburia* and *Faecalibacterium* species are SCFAs-producing bacteria and also possess antiinflammatory properties (Zhang et al., 2013; Remely et al., 2014). These microbial-produced SCFA metabolites such as butyrate are important in epigenetic modulations, such as HDAC inhibition (Donohoe et al., 2014). As aforementioned, butyrate also plays an essential role in the differentiation of T_reg_ cells through acetylation of noncoding regions of the *Foxp3* locus (Furusawa et al., 2013). Various other studies also investigated the relationship between alterations in gut microbiota in type 2 diabetes and their associated metabolic and epigenetic mechanisms which are detailed in **Table 3**. Over the past years, it has become increasingly apparent that epigenetic modifications such as DNA methylation are associated with type 2 diabetes. Genome-wide association studies have revealed the correlation of single nucleotide polymorphisms of type 2 diabetes with defect in secretion of insulin, which corresponds to abnormality in pancreatic islet cells (Ruchat et al., 2009). Therefore, DNA methylation levels of CpG sites and the transcriptome in pancreatic islets were analyzed and compared between type 2 diabetes patients and normal individuals (Dayeh et al., 2014). This study detected 1,649 CpG sites and 853 genes, including *KCNQ1*, *TCF7L2,* and *FTO,* with differential DNA methylation in islets from type 2 diabetes patients (Dayeh et al., 2014). The findings also include 102 genes exhibiting differential DNA methylation as well as differential gene expression in islets from type 2 diabetes. Those genes include *CDKN1A*, *PDE7B*, *SEPT9,* and *EXOC3L2*, which were found to be key genes in regulating insulin secretion in β-cells and glucagon secretion in pancreatic α-cells (Dayeh et al., 2014). This study suggests that epigenetic mechanisms may contribute significantly to the regulation and developmental process of insulin resistance and type 2 diabetes *via* pancreatic cells.

**Table 3:** Interplay between gut microbiota, metabolic mechanisms, epigenetic modifications, and type 2 diabetes.

Study design	Method	Gut microbiota profile	Associated metabolic mechanisms	Association between microbiota and epigenetic modifications	References
Metagenome-wide association study on fecal samples of 345 Chinese individuals	16S rRNA gene sequencing	Less levels of *Roseburia* species and *Faecalibacterium prausnitzii* were found in type 2 diabetes patients Elevated levels of *Escherichia coli* (Proteobacteria phylum) in the type 2 diabetes	These are SCFAs – producing bacteria and possess antiinflammatory properties. Also, *Faecalibacterium prausnitzii* is also a metabolic modulator *Escherichia coli* is a proinflammatory and opportunistic bacteria	These produced SCFAs such as butyrate, causes HDAC inhibition. This resulting HDAC inhibition led to downregulation of mRNA expression of *p21* and *p27* (cell cycle regulators) and may ameliorate transcription of *Fas* (a proapoptotic gene) Transplantation of normal mice microbiota *with E. coli* into germ-free mice led to differential lncRNA changes	(Qin et al., 2012; Karlsson et al., 2013) (Zhang et al., 2013; Remely et al., 2014) (Donohoe et al., 2014) (Liang et al., 2015)
Sixty type 2 diabetes patients were recruited in Japan. They were divided into two groups- placebo or transglucosidase for 12 weeks. In addition, fecal bacterial composition was compared with 10 healthy participants	T-RFLP analysis	Significant decline in the level of *Clostridium* and rise in the levels of *Lactobacillales* and *Bifidobacterium* in type 2 diabetes patients as compared with healthy controls	*Clostridium* are SCFAs – producers and involved in maintenance of gut immune homeostasis	*Clostridium* were associated with dephosphorylation of H3 histone and deacetylation of H4 histone, which may led to decreased transcription of major genes involved in immunity	(Sasaki et al., 2013) (Hamon et al., 2007)
145 women of European origin, with normal glucose tolerance, impaired glucose tolerance, or type 2 diabetes	Shotgun sequencing	Increase in the levels of *Lactobacillus* species in the type 2 diabetes group	Immunomodulating properties and some species of *Lactobacillus* are probiotic It also protects host from pathogenic microorganisms by production of lactic acid in vagina	*Lactobacillus* involved with upregulation of *miR-21-5p* in intestinal epithelial cells	(Karlsson et al., 2013) (Voravuthikunchai et al., 2006) (Nakata et al., 2017)
The investigation was done on three groups- insulin-dependent type 2 diabetes patients on glucagon like 1 peptide therapy (n = 24), obese individuals (n = 14) and lean individuals (n = 18)	High-throughput sequencing and fragment-length polymorphism analysis	Abundance of *Bacteroides vulgatus* significantly increased in type 2 diabetes patients	*Bacteroides vulgatus* exhibits proinflammatory properties	*Bacteroides fragilis* toxin have been known to cause colonic hyperplasia, colitis and tumor progression by signal transduction and activation of transcription 3 and a proinflammatory response	(Marlene et al., 2016) (Wu et al., 2009)

**Figure 3:**
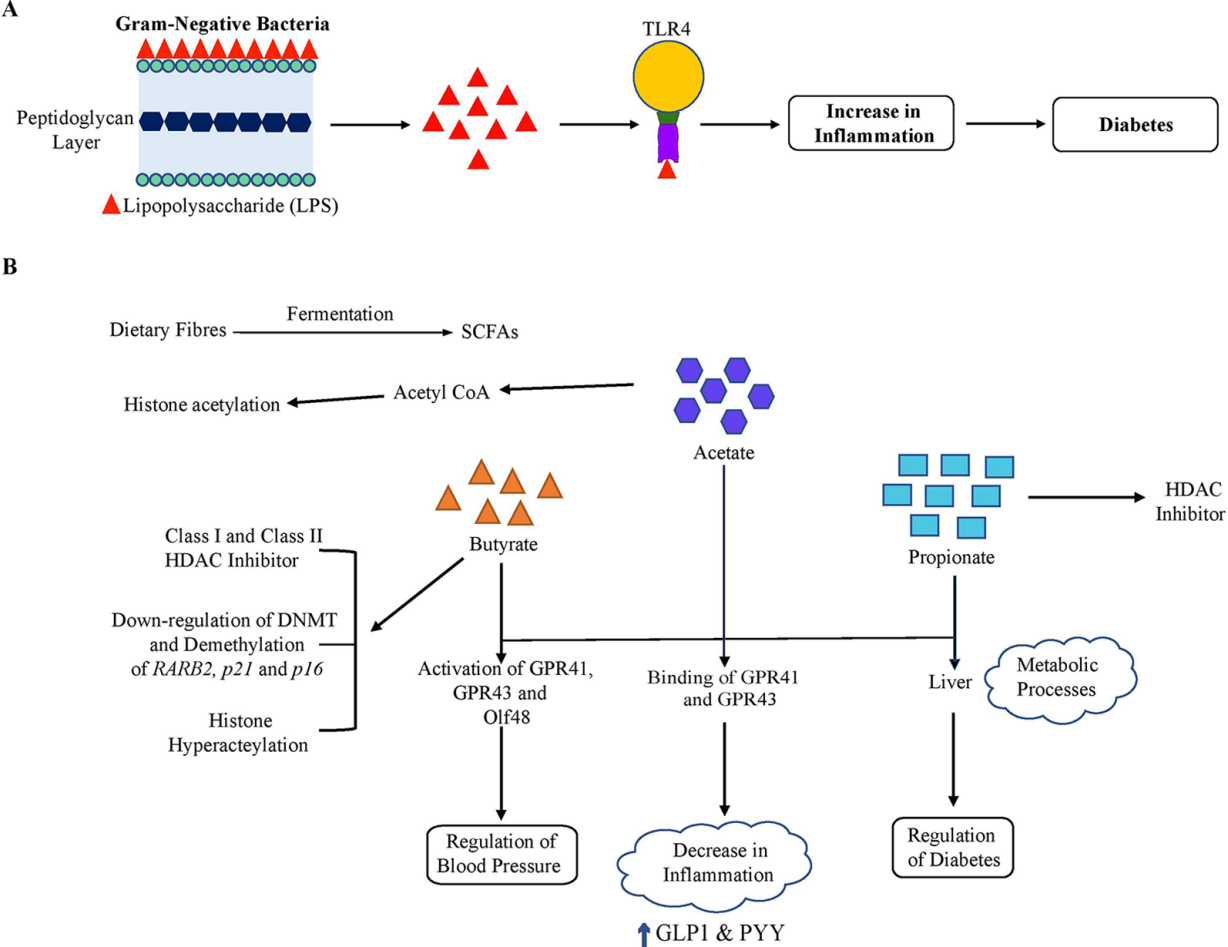
Gut microbiome and host metabolism. The gut microbiome may alter host metabolism through many mechanisms. Of these, two important mechanisms are illustrated. **(A)** Lipopolysaccharide (LPS). LPS originates from the outer membrane of Gram-negative bacteria and binds to Toll-like receptor 4 (TLR4), which results in low-grade inflammation and thus a decline in insulin sensitivity. **(B)** Short-chain fatty acids (SCFA). The gut microbiota ferments dietary fibers to SCFAs, including acetate, butyrate, and propionate. Acetate and propionate participate in essential metabolic processes such as gluconeogenesis and lipogenesis in the liver. In addition, SCFAs bind to the G protein-coupled receptors GPR41 and GPR43 resulting in various effects depending on the cellular types affected. In immune cells, this signaling led to a decrease in the inflammation and resulted in an increase in GLP1 and PYY levels in enteroendocrine L-cells, which improve insulin sensitivity overall. Also, these SCFAs activate GPR41, GPR43, and Olfr78 expressed in the kidney. Olfr78 induces SCFA-mediated release of renin which leads to rise in blood pressure. On the other hand, GPR43 resists this change in blood pressure by vasodilatory action. SCFAs are involved in the induction of epigenetic alterations. Butyrate is a known class I and class II HDAC inhibitor. Butyrate can also affect DNA methylation and demethylation of some tumor suppressor genes (*RARB2, p21,* and *p16*) and is involved in acetylation of histone H3. Propionate is also a contributor to HDAC inhibition. In addition, acetate has been found to be involved in increasing histone acetylation *via* transferring an acetyl group from acetyl-CoA.

The link between the gut microbiome and host metabolism is also shown in **Figure 3**. The gut microbiome can modulate the host metabolism in multiple ways and may result in insulin resistance and type 2 diabetes. First, with an abundance of gram-negative bacteria such as *Escherichia coli,* it has been hypothesized that the detachment of lipolysaccharide (LPS), the outer layer of gram-negative bacteria, may induce proinflammation in type 2 diabetes as well as in obesity (Allin et al., 2015). Secreted lipopolysaccharide binds with Toll-like receptor 4 (TLR4) and activates proinflammatory immune pathways, which leads to low-grade inflammation and further decreased insulin sensitivity (Cani et al., 2007). The decline in microbial gene richness was also found to be associated with low-grade inflammation (Cotillard et al., 2013). Second, three main types of SCFAs, butyrate, propionate, and acetate, produced from the fermentation of dietary fibers by gut bacteria, can influence glucose and energy metabolism of the host. Acetate and propionate are known to participate in essential metabolic processes such as glyconeogenesis and lipogenesis in the liver. Butyrate acts as an essential energy substrate for colonic mucosal cells and exhibits a positive effect on insulin sensitivity (Gao et al., 2009). These SCFAs bind to GPR41 and GPR43, leading to various effects depending on the types of cells affected (Brahe et al., 2013). In immune cells, the binding of SCFAs with GPRs results in lesser development of inflammation. However, it contributes to an increase in the secretions of GLP1 and PYY from L-cells (an enteroendocrine cell) in the colon, which improves insulin sensitivity (Lin et al., 2012). It has been demonstrated that enhancing the levels of GLP-1 by altering the composition of the gut microbiota with exposure to antibiotics leads to improved glucose tolerance, insulin resistance and a rise in beneficial metabolites such as succinic acid (Hwang et al., 2015). In addition, SCFAs are found to regulate glucose metabolism by intestinal gluconeogenesis.

SCFAs also play a crucial role in the regulation of blood pressure by renin secretion as also shown in **Figure 2**. The SCFAs induce activation of GPR41, GPR43, and Olfr78, an olfactory receptor expressed in the kidney. Olfr78 also participates in the secretion of renin, induced by SCFA from afferent arterioles that lead to a rise in the blood pressure. High blood pressure is a feature of metabolic syndrome, which includes metabolic alterations such as obesity, hypertension, glucose intolerance, dyslipidemia, diabetes, etc (Schillaci et al., 2004; Lin et al., 2009). However, GPR43 resists this change in blood pressure by vasodilatory action (Pluznick et al., 2013; Pluznick, 2014). In adipocytes, binding of SCFA with GPR43 contributes to enhanced metabolism by restricting fat accumulation in adipose tissue (Kimura et al., 2013). Thus, microbial-produced SCFAs may beneficially regulate glucose metabolism which correlate with a low risk of diabetes and also regulate the blood pressure.

## Conclusion

The gut microbiome is regulated by multiple factors such as diet, environment, genetics and epigenetics. Several studies have implicated the interactions of the gut microbiome with the host epigenome, which shows a potential role of the gut microbiome in the regulation of host metabolism. The modulation by the gut microbiome to the host epigenome may be due to direct and frequent contact with the host as well as due to various microbial-derived metabolites produced in the gut. For instance, SCFAs produced in the gut, predominately acetate, butyrate, and propionate, interact with cell surface receptors and with the epithelial and submucosal layers of the colon thereby influencing obesity and diabetes outcome. Although SCFAs are crucial to alter the epigenetic processes of the host through DNA methylation as well as histone modifications, further studies are needed to elucidate the underlying molecular mechanisms and their biological properties in the hosts. For example, which bacterial species have symbiotic relationships, and in what manner do their metabolites participate in specific metabolic processes in humans? Hence, future studies focusing on specific gut microbial metabolites that affect the host epigenome will give new insights into the health and metabolic disease of humans. In addition, the studies focusing on epigenetic mechanisms of the gut microbiome and its influence in obesity and diabetes are emerging. This could hold a promising future in uncovering novel therapeutic mechanisms that may restore the altered intestinal microbiome to a healthy condition and assist in the prevention and treatment of obesity as well as diabetes.

## Author Contributions

MS and TT conceived of the review article and participated in all drafts of the manuscript. MS wrote the first draft of the manuscript with guidance from TT. YL and MLS participated in editing of several drafts. TT performed final editing and approval of the manuscript. All authors read and approved the final draft.

## Funding

This work was supported in part by grants from the National Institute of Health (NCI R01CA178441, NCI R01CA204346, NCCIH K01AT009373, and NIDDK P30DK056336).

## Conflict of Interest

The authors declare that the research was conducted in the absence of any commercial or financial relationships that could be construed as a potential conflict of interest.

## Abbreviations

SCFA, Short-chain fatty acid; TMA, Trimethylamine; FMO, Flavin monooxygenase; TMAO, Trimethylamine-*N*-oxide; ncRNA, noncoding RNA; HFD, High fat diet; miRNA, microRNA; siRNA, short-interfering RNA; piRNA, piwi-intreacting RNA; lncRNA, long noncoding RNA; CpG, cytosine-guanine dinucleotide-rich; DNMT, DNA methyltransferase; SAM, S-adenosylmethionine; HAT, Histone acetyltransferase; HDAC, Histone deacetylase; Treg, regulatory T cells; ROS, Reactive oxygen species; LPS, Lipolysaccharide; TLR4, Toll-like receptor 4; GPR, G-protein coupled receptor.
